# High-Throughput Analysis of Astrocyte Cultures Shows Prevention of Reactive Astrogliosis by the Multi-Nutrient Combination Fortasyn Connect

**DOI:** 10.3390/cells11091428

**Published:** 2022-04-22

**Authors:** Aina Badia-Soteras, Janneke de Vries, Werner Dykstra, Laus M. Broersen, Jan Martin Verkuyl, August B. Smit, Mark H. G. Verheijen

**Affiliations:** 1 Department of Molecular and Cellular Neurobiology, Center for Neurogenomics and Cognitive Research, Faculty of Earth and Life Sciences, Vrije Universiteit, 1081 HV Amsterdam, The Netherlands; a.badiasoteras@vu.nl (A.B.-S.); jwdevries93@gmail.com (J.d.V.); w.dykstra@umcutrecht.nl (W.D.); guus.smit@vu.nl (A.B.S.); 2 Danone Nutricia Research, 3584 CT Utrecht, The Netherlands; laus.broersen@danone.com (L.M.B.); martin.verkuijl@danone.com (J.M.V.)

**Keywords:** method, astrocytes, reactive astrogliosis, nutritional intervention

## Abstract

Astrocytes are specialized glial cells that tile the central nervous system (CNS) and perform numerous essential functions. Astrocytes react to various forms of CNS insults by altering their morphology and molecular profile, through a process known as reactive astrogliosis. Accordingly, astrocyte reactivity is apparent in many neurodegenerative diseases, among which one is Alzheimer’s disease (AD). Recent clinical trials on early-stage AD have demonstrated that Fortasyn Connect (FC), a multi-nutrient combination providing specific precursors and cofactors for phospholipid synthesis, helps to maintain neuronal functional connectivity and cognitive performance of patients. Several studies have shown that FC may act through its effects on neuronal survival and synaptogenesis, leading to reduced astrocyte reactivity, but whether FC can directly counteract astrocyte reactivity remains to be elucidated. Hence, we developed an in vitro model of reactive astrogliosis using the pro-inflammatory cytokines TNF-α and IFN-γ together with an automated high-throughput assay (AstroScan) to quantify molecular and morphological changes that accompany reactive astrogliosis. Next, we showed that FC is potent in preventing cytokine-induced reactive astrogliosis, a finding that might be of high relevance to understand the beneficial effects of FC-based interventions in the context of neurodegenerative diseases.

## 1. Introduction

Astrocytes are the most abundant cells in the brain and perform a vast array of functions that are essential for the central nervous system (CNS), e.g., maintaining cellular homeostasis [1,2], providing metabolic support to neurons [3], and modulating synaptic transmission [4,5,6]. In addition, astrocytes are important players in the diseased CNS; they respond to many forms of CNS insults by undergoing a variety of molecular, cellular, and functional changes. Such responses are known as astrocyte reactivity or reactive astrogliosis. In fact, reactive astrogliosis is a finely tuned dynamic process that is modulated in a context-specific manner by a wide variety of signaling molecules and ranges from mild to severe, depending on whether these changes are reversible or permanent [7,8,9,10]. This reactive state can be induced by a wide number of extracellular molecules (e.g., purines, transmitters, and pro-inflammatory cytokines) or neurodegeneration-associated molecules (e.g., amyloid beta) [8,11]. Moreover, reactive astrocytes exhibit specific transcriptomic, morphological, and functional changes; they upregulate inflammatory mediators, reactive oxygen species, and neurotrophic factors [12,13,14,15]; undergo cell hypertrophy due to the upregulation of intermediate filaments, e.g., glial fibrillary acidic protein (GFAP), Nestin, and vimentin [9,16,17,18]; alter intracellular calcium signaling (e.g., S-100β) [18,19]; and provide less support in synapse formation and function [14,20,21]. Importantly, reactive astrocytes have been described to play a dual role, as they can both strengthen a pro-inflammatory environment, contributing to neuronal dysfunction, and exert neuroprotective actions, promoting CNS recovery and repair [14,18,22,23,24]. Both attenuating neurotoxic astrocytic responses as well as supporting neuroprotective astrocytes functions are key therapeutic targets for clinical interventions in the context of CNS disorders.

Preclinical studies have shown that reactive astrogliosis is a potential underlying factor in many neurodegenerative diseases, such as Alzheimer’s disease (AD) and Parkinson’s disease (PD) [25,26,27,28], and early glial changes have been suggested to play an important role in early pathology [29]. However, little is known about glia-related mechanisms in the context of disease, and because in most cases the development of specific drugs is mainly focused on direct effects on neurons rather than glial cells, more research is needed to explore the direct role of glial reactivity in disease progression.

Interestingly, a novel multi-nutrient intervention that recently was shown to improve neuronal survival and postsynaptic maturation in a neuron-astrocyte co-culture [30] has now been shown to slow decline in clinical and other measures related to cognition, function, brain atrophy, and disease progression in prodromal AD [31,32]. Previously, this nutrient combination, called Fortasyn Connect (FC), comprising docosahexaenoic acid (DHA); eicosapentaenoic acid (EPA); uridine monophosphate (UMP); choline; phospholipids; folic acid; vitamins B12, B6, C, and E; and selenium improved memory performance in patients with mild AD [33,34]. In addition, de Waal and colleagues reported that FC preserved functional connectivity and counteracted the progressive network disruption described in AD [35]. Similarly, in a mouse model of AD, dietary intervention with FC enhanced neuroprotective mechanisms supporting gray and white matter integrity [36]. The underlying mechanisms of these rescue effects of FC may involve the regulation of glial responses, as FC supplementation improved functional recovery that coincided with a reduced reactive astrogliosis after spinal cord injury [37]. However, whether FC is able to directly target reactive astrogliosis remains to be determined. Thus, our study is focused on finding a potential role of FC in the attenuation of reactive astrogliosis. For this, we designed and optimized an automated high-throughput assay that enabled us to reproducibly quantify reactive astrogliosis from primary hippocampal astrocytes. We found that the presence of the pro-inflammatory cytokines tumor necrosis factor-alpha (TNF-α) and interferon-gamma (IFN-γ) was sufficient to induce an astrocyte reactive state and that this could be prevented by the addition of the specific multi-nutrient combination FC. 

## 2. Materials and Methods

### 2.1. Primary Hippocampal Astrocyte Culture

All animal procedures were performed according to the guidelines of the Central Committee for Animal Experiments (CCD) and the Animal Welfare Body (IVD) of the VU University Amsterdam (The Netherlands). Wild-type (WT) C57BL/6J mice were obtained from Charles River and bred in the animal facility of the VU University Amsterdam. Primary mouse astrocytes were obtained from postnatal day 1 (P1) C57Bl/6J mice. In brief, hippocampi were isolated from P1 mice, cleared of meninges, and collected in ice-cold Hanks Buffered Salt Solution (HBSS; Sigma-Aldrich, St. Louis, MO, USA) buffered with 7 mM HEPES (pH 7.4; Life Technologies, Amsterdam, The Netherlands). The tissue was digested with 0.25% trypsin for 20 min at 37 °C. Subsequently, a blocking solution was added, consisting of DMEM (Dulbecco’s modified Eagle’s medium + Glutamax; Thermo Fisher, Waltham, MA, USA) supplemented with a non-essential amino acid solution (Sigma-Aldrich, St. Louis, MO, USA), 1% penicillin–streptomycin (Life Technologies, Amsterdam, The Netherlands), and 20% fetal bovine serum (FBS; Thermo Fisher, Waltham, MA, USA). The cell suspension was centrifuged at 1200 rpm for 10 min at room temperature (RT) and the pellet resuspended. Then, 6000 cells/well were plated in DMEM 10% FBS on a coated 96-well plate kept in a 37 °C/5% CO_2_ incubator. Coating of the plates was done 1 day prior to plating, and it included poly-D-lysine/laminin (PDL/laminin; 20 μm/mL; Sigma-Aldrich, St. Louis, MO, USA), tenascin-c (5 μm/mL; Sigma-Aldrich, St. Louis, MO, USA), or fibronectin (20 μm/mL; Sigma-Aldrich, St. Louis, MO, USA). At 5 days in vitro (DIV5), the medium was replaced by (1) DMEM + 10% FBS, (2) DMEM + 0.1% FBS, or (3) neurobasal medium (NBM) (Thermo Fisher, Waltham, MA, USA), supplemented with 1.8% HEPES (Invitrogen, Carlsbad, CA, USA) and either B27 (1:50; Thermo Fisher, Waltham, MA, USA) or N2 (1:100; Thermo Fisher, Waltham, MA, USA) [38]. All media contained 5 mM Glutamax (Invitrogen, Carlsbad, CA, USA) and 0.1% penicillin–streptomycin (Invitrogen, St. Louis, MO, USA). Immuno-fluorescence analysis of astrocyte, oligodendrocyte, microglia, and neuron-specific markers (GFAP/S-100β, Aldh1l1, Olig2, Iba-1 and NeuN, respectively) revealed that 98% of the cells present in the culture were astrocytes, 1.5% microglia, and 0.04% oligodendrocytes (Figure S1).

### 2.2. Cytokine and FC Treatment 

**Figure 1:**
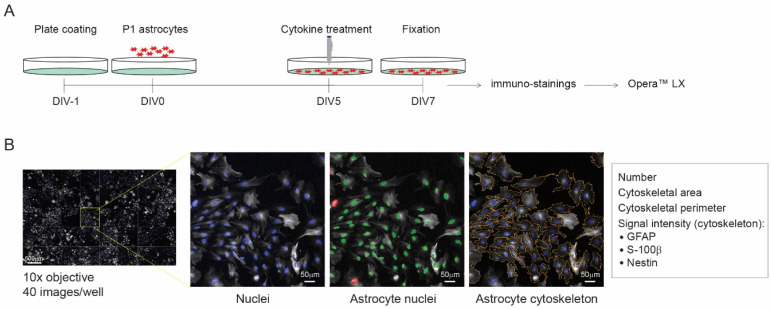
AstroScan. (**A**) Experimental design of the cytokine-induced reactive astrogliosis model. (**B**) Left: Overview image showing 40 fields per well (96-well plate) taken with a 10× objective from the Opera™ LX (PerkinElmer) automated confocal microscopy. Close-up: Example image of one field. Columbus software was used to identify and select single astrocyte nuclei (based on DAPI intensity, size, and roundness) as well as the astrocyte cytoskeletal area and perimeter (based on GFAP immunostaining). GFAP, Nestin, and S-100β protein levels were quantified for selected astrocytes.

To reliably induce a reactive phenotype, we treated astrocytes with two well-studied pro-inflammatory cytokines, tumor necrosis factor-alpha (TNF-α) and interferon-gamma (IFN-γ), that have been shown to be involved in the initiation and persistence of glial activation in vitro and in vivo [14,39,40,41] and do this synergistically [12,42,43]. Hence, 10 ng/mL of TNF-α (R&D Systems, Minneapolis, MN, USA, Cat # 580102,) and IFN-γ (BioLegend, San Diego, CA, USA, Cat # 585-IF-CF) were added to the astrocyte culture at DIV5 (Figure 1A) [42]. The following components were present in the FC cocktail: docosahexaenoic acid (DHA), eicosapentaenoic acid (EPA), uridine, choline chloride, vitamin B6, vitamin B12, vitamin B9 (folic acid), phosphatidylcholine (PC), vitamin C (ascorbic acid), vitamin E, and selenium (sodium selenite). The FC nutrients were dissolved in the following solvents: DHA and EPA in absolute ethanol and FBS (EtOH 1:5 FBS), vitamin B in 1 M HCl, and PC and vitamin E in absolute EtOH, folic acid in 1 M NaOH, and all the other nutrients in demineralized water. Each FC component was dissolved in 1:50 HBSS and stored at −80 °C until further use. Prior to supplementation, the nutrients were combined with a fresh culture medium and added to the cells in the final dilutions of 0.2× FC (1:5000) or 0.05× FC (1:20,000); see Table 1. For control conditions, solvents were added to the culture medium in corresponding concentrations depending on FC supplementation. Astrocytes (6000 cells/well) were seeded on a fibronectin culture matrix and cultured on DMEM + 10% FBS until DIV5, when the culture medium was replaced with NBM + N2 containing 10 ng/mL of TNF-α and 10 ng/mL of IFN-γ together with 0.2× or 0.05× FC, respectively.

### 2.3. Immunofluorescence

Cells were fixed with 4% PFA/4% sucrose for 20 min at room temperature and washed 3 times with 0.1 M phosphate buffered saline (PBS) pH 7.4 for 10 min. Subsequently, a blocking solution consisting of 3% bovine serum albumin (Sigma-Aldrich, St. Louis, MO, USA) and 0.2% Triton X-100 in 0.1 M PBS was added to the cells and kept for 1 h at RT. Cells were incubated overnight with primary antibodies in the blocking solution at 4 °C, followed by 3 washing steps with 0.1 M PBS, and incubated for 1 h at RT with the corresponding secondary antibodies. Subsequently, the cells were washed 3 times with 0.1 M PBS and incubated with Baxter water (Baxter, Deerfield, MA, USA) for 10 min, followed by DAPI incubation (1:20,000; Molecular Probes, Eugene, OR, USA) for 10 min. Lastly, the cells were washed again with Baxter water and kept in PBS at 4 °C until further analysis. The primary antibodies used were: rabbit anti-GFAP (1:1000; Agilent Dako, Santa Clara, CA, USA, Cat # Z033401), mouse anti-S-100β (1:1000; Sigma-Aldrich, St. Louis, MO, USA, Cat # S2532), mouse anti-Nestin (1:700; BD Biosciences, San Jose, CA, USA, Cat # 611658), mouse anti-Ki67 (1:100; Santa Cruz, Heidelberg, Germany, Cat # sc-23900), mouse anti-NFκB (p65) (1:250; Santa Cruz, Heidelberg, Germany, Cat # sc-8008), rabbit anti-Aldh1l1 (1:1000, Abcam, Cambridge, UK, Cat # ab190298), rabbit anti-Iba-1 (1:500, Abcam, Cambridge, UK, Cat # ab17887), and goat anti-Olig2 (1:500, R&D Systems, Minnesota, US, Cat # AF2418). All secondary antibodies were used at 1:400 (Thermo Fisher Scientific, The Netherlands): rabbit Alexa 488 (Cat # A-11008), rabbit Alexa 568 (Cat # A-11011), mouse Alexa 568 (Cat # A-11004), mouse Alexa 647 (Cat # A-21235), and goat Alexa 488 (Cat # A-21467). 

### 2.4. High-Throughput Screening

Images were taken with an Opera™ LX (PerkinElmer, Waltham, MA, USA) automated confocal microscopy system and analyzed with Columbus software (version 2.5.2, Perkin Elmer). In all, 40 images/well were taken in a randomized manner with a 10× magnification microscope. 

### 2.5. Analysis of Reactive Astrogliosis

To evaluate cytokine-induced astrocyte reactivity, the following parameters were analyzed (per astrocyte): immunofluorescence intensity of marker proteins, intermediate filament cytoskeletal area, and perimeter (based on GFAP staining). The analysis was performed as follows: (1) Nuclei detection, based on the intensity of the DAPI signal. (2) Identification of astrocyte nuclei, based on size and roundness from the previously selected nuclei. This step is used to discard debris and other cell types. (3) Measurement of the intensity of the intermediate filaments GFAP and Nestin and the calcium binding protein S-100β for selected astrocytes. (4) Determination of morphological changes analyzing the perimeter and area of the astrocyte cytoskeleton based on GFAP immuno-staining (Figure 1B). To determine NF-κB nuclear translocation, the NF-κB cytoplasm intensity was subtracted from the NF-κB nucleus intensity.

### 2.6. Viability/Cytotoxicity Assay

Astrocytes were isolated from P1 mice as indicated previously and seeded at a desired density of 6000 cells/well. At DIV5, astrocytes were stimulated with 10, 20, or 50 ng/mL of TNF-α and IFN-γ. Two days later, cell survival was assessed with a Live/Dead^TM^ Viability/Cytotoxicity kit (Thermo Fisher, Waltham, MA, USA). The percentage of viable cells was calculated dividing the number of living cells (green fluorescence) by the total number of cells (per well).

### 2.7. SDS-PAGE Immunoblotting

Astrocytes (95,000 cells/well, 24 well-plate) were lysed 48 h after treatment with 5× Laemmli buffer. Samples were incubated at 90 °C for 5 min, and proteins were separated on SDS-polyacrylamide gels. After electrophoresis, gels were transferred on to a PVDF membrane overnight at 40 V. Membranes were blocked with 5% non-fat milk (Sigma-Aldrich, St. Louis, MO, USA), followed by primary antibody incubation over night at 4 °C. Next, HRP-conjugated secondary antibodies were added (Agilent Dako, Santa Clara, CA, USA) to the membranes and kept for 1 h at RT. Finally, membranes were scanned with Femto ECL Substrate (Thermo Fisher Scientific, Waltham, MA, USA) using the Odyssey Fc system (LI-COR Bioscience, Lincoln, NE, USA). Images were quantified with Image Studio software (version 2.0.38). The following primary antibodies were used: rabbit anti-GFAP (1:1000; Agilent Dako, Santa Clara, CA, USA), mouse anti-Vimentin (1:200, Santa Cruz, Heidelberg, Germany), and mouse anti-actin (1:10,000, Sigma-Aldrich, St. Louis, MO, USA). Total protein levels for GFAP and Vimentin were normalized to actin.

### 2.8. Data Collection and Statistical Analysis

**Figure 2:**
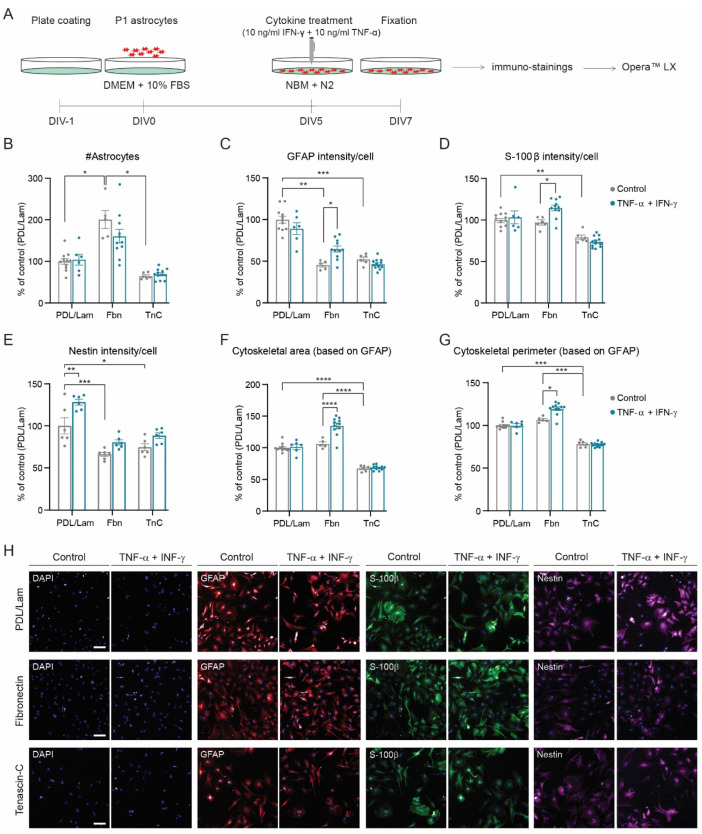
Cytokine-induced reactive astrogliosis in vitro: the effect of plate coating. (**A**) Experimental design. Analysis of (**B**) number of astrocytes, (**C**) GFAP intensity per cell, (**D**) S-100β intensity per cell, (**E**) Nestin intensity per cell, (**F**) cytoskeletal area (based on GFAP intensity), and (**G**) cytoskeletal perimeter (based on GFAP intensity). (**H**) Representative images of DAPI (blue), GFAP (red), S-100β (green), and Nestin (magenta) for each condition. PDL/Lam (PDL/Laminin), Fbn (fibronectin), and Tnc (tenascin-c). Data were normalized to PDL/Lam unstimulated. N = 6–10 wells/condition; n = 7804 and n = 4438 astrocytes analyzed for PDL/Lam-unstimulated and PDL/Lam-stimulated condition, respectively; n = 7055 and n = 12,533 for Fbn condition; and n = 2790 and n = 5967 for TnC condition. Data are presented as the mean ± SEM. Two-way ANOVA with a Bonferroni post hoc test: * *p* < 0.05, ** *p* < 0.01, *** *p* < 0.001, and **** *p* < 0.0001. Scale bar: 100 µm.

**Figure 3:**
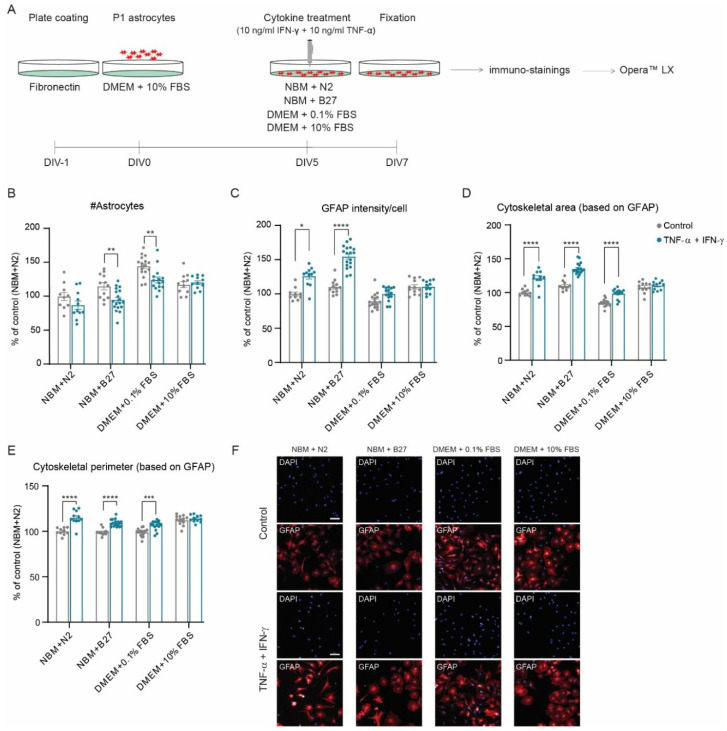
Cytokine-induced reactive astrogliosis in vitro: the effect of culture medium. (**A**) Experimental design. Analysis of (**B**) number of astrocytes, (**C**) GFAP intensity per cell, (**D**) cytoskeletal area (based on GFAP intensity), and (**E**) cytoskeletal perimeter (based on GFAP intensity). (**F**) Representative images of DAPI (blue) and GFAP (red) for each condition. Data were normalized to NBM + N2 unstimulated (N = 10–18 wells/condition; n = 14,298 and n = 10,963 astrocytes analyzed for NBM + N2 unstimulated and stimulated condition, respectively; n = 10,823 and n = 25,485 for NBM + B27 condition; n = 37,021 and n = 27,801 for DMEM + 0.1% FBS condition; and n = 21,259 and n = 19,921 for DMEM + 10% FBS condition). Data are presented as the mean ± SEM. Two-way ANOVA with a Bonferroni post hoc test: * *p* < 0.05, ** *p* < 0.01, *** *p* < 0.001, and **** *p* < 0.0001. Scale bar: 100 µm.

For statistical power and randomization purposes, 40 images/well were taken from 6–12 wells/condition, which were spread arbitrarily throughout the plate (96-well plate). The outlier removal was performed using GraphPad Prism 9.0 (ROUT method; Q = 1%). All data sets were tested for normality using the Shapiro–Wilk test. Statistical analyses were performed using one-way ANOVA and two-way ANOVA, with a Bonferroni test for post hoc analysis. All experiments were replicated at least 3 times (except Figure 2 and Figure 3; 2 times).

## 3. Results

To evaluate the effect of FC on reactive astrogliosis, we first developed an in vitro model by establishing the optimal culture conditions to consistently trigger a reactive phenotype. Hence, we tested different culture conditions that are known to affect astrocyte reactivity: plate coating, culture media, and cytokine concentration. Next, we used a high-throughput analysis to evaluate how astrocyte morphology and reactive astrogliosis markers (GFAP, S-100β, and Nestin) were affected under these conditions (Figure 1) [7,8].

### 3.1. Development of a Reactive Astrogliosis In Vitro Model; the Effect of the Plate Coating

Previous studies have shown that culture matrix composition affects the reactive phenotype of astrocytes as well as their response to cytokines [44,45]. Hence, we investigated astrocytic responses on different culture matrix conditions, PDL/laminin, fibronectin, and tenascin-c, and determined the effect of two pro-inflammatory cytokines, TNF-α and IFN-γ [42] (Figure 2A–H). 

The number of astrocytes was higher when cultured on fibronectin than when cultured on PDL/Laminin and tenascin-c (Figure 2B). Protein levels for GFAP were lowest for astrocytes cultured on fibronectin and tenascin-c (Figure 2C,H), whereas S-100β and Nestin protein levels were lowest for astrocytes cultured on tenascin-c (Figure 2D,E,H). In addition, culture matrix composition also influenced astrocyte morphology: astrocytes cultured on tenascin-c had a smaller cytoskeletal area and perimeter (based on GFAP immunostaining) compared to astrocytes cultured on fibronectin and PDL/Laminin (Figure 2F–H). Furthermore, astrocyte stimulation with TNF-α and IFN-γ was most successful for astrocytes cultured on fibronectin: GFAP and S-100β protein levels were increased (Figure 2C,D,H), as well as the astrocyte cytoskeletal area and perimeter (Figure 2F–H), whereas the astrocyte number did not change significantly (Figure 2B). Additionally, we tested whether cytokine treatment for 24 h was sufficient to induce a reactive state. Indeed, we also observed reactive astrogliosis, even though it was less profound than at 48 h (Figure S2A–F). Therefore, we continued our studies with the 48 h time point. 

Taken together, culture matrix composition significantly influences astrocyte reactivity, with fibronectin being the most suitable coating for cytokine-induced reactive astrogliosis.

### 3.2. Development of a Reactive Astrogliosis In Vitro Model; the Effect of Culture Media

Next, the effect of different culture media on cytokine-induced astrocyte reactivity was determined (Figure 3A–F), considering that components present in serum (e.g., albumin) or serum-free supplements may influence the reactive state of astrocytes [8,15,46,47,48,49].

Cytokine treatment significantly decreased the number of astrocytes in NBM + B27 and DMEM + 0.1% FBS media (Figure 3B), while not in NBM + N2 or DMEM + 10% FBS. Moreover, the induction of reactive astrogliosis was most optimal for astrocytes cultured in NBM + B27 and NBM + N2 media (Figure 3C–F). Astrocytes cultured in DMEM + 10% FBS did not show any reactive response to cytokines (Figure 3C–F), remarkably. As the main purpose for developing a cytokine-induced reactive astrogliosis model was to evaluate the anti-inflammatory properties of the specific-nutrient combination FC, which contains 11 phospholipid precursors and cofactors, the most minimal medium, NBM + N2, was chosen to assess the putative effects of FC on reactive astrogliosis.

### 3.3. Differential Effect of TNF-α and IFN-γ Concentration on the Development of a Reactive Astrogliosis Model

**Figure 4:**
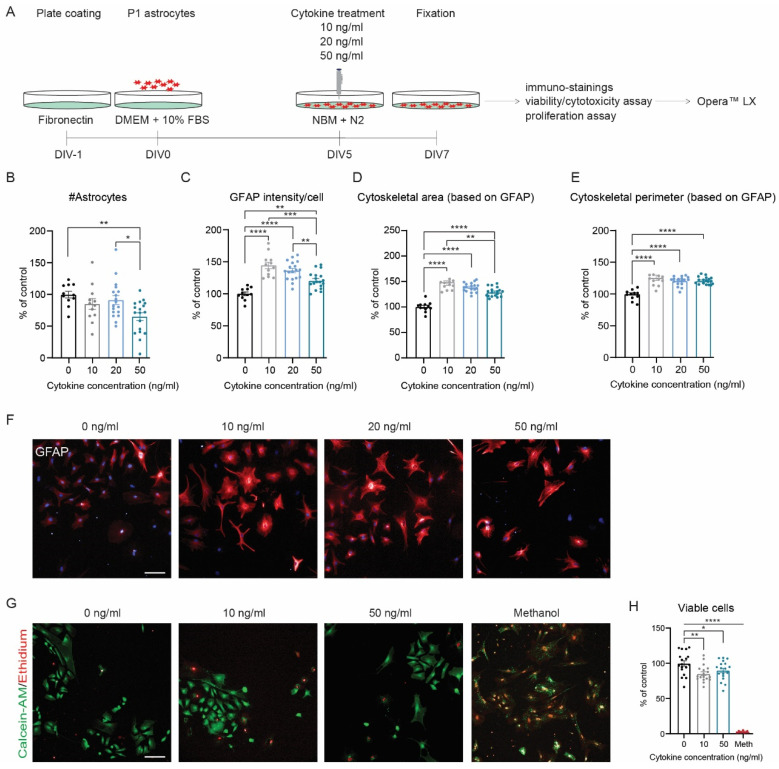
Cytokine-induced reactive astrogliosis in vitro: optimization of cytokine concentration. (**A**) Experimental design. Analysis of (**B**) number of astrocytes, (**C**) GFAP intensity, (**D**) cytoskeletal area (based on GFAP intensity), and (**E**) cytoskeletal perimeter (based on GFAP intensity). (**F**) Representative images of GFAP (red) and DAPI (blue). N = 10–18 wells/condition; n = 24,701 (control), n = 22,980 (10 ng/mL), n = 36,734 (20 ng/mL), and n = 26,294 (50 ng/mL) astrocytes analyzed. (**G**) Representative images of alive (green) and dead (red) astrocytes upon incremental cytokine stimulation. (**H**) Analysis of the percentage of viable cells. N = 16–18 wells/condition; n = 4377 (control), n = 2876 (10 ng/mL), n = 1906 (50 ng/mL), and n = 2304 (methanol) astrocytes analyzed. Data were normalized to the non-stimulated condition. Data are presented as the mean ± SEM. One-way ANOVA with a Bonferroni post hoc test: * *p* < 0.05, ** *p* < 0.01, *** *p* < 0.001, and **** *p* < 0.0001. Scale bar: 100 µm.

To establish the optimal concentration of cytokines to induce a reactive state, we tested three different concentrations for both TNF-α and IFN-γ (Figure 4A–F). All three concentrations of cytokines induced a reactive phenotype: increased GFAP protein levels and enlarged astrocyte cytoskeleton (Figure 4C–E), with 10 and 20 ng/mL being more potent than 50 ng/mL. In addition, the number of astrocytes was slightly affected at 10 ng/mL, while it significantly decreased at 50 ng/mL (Figure 4B). Next, we tested whether the loss of astrocytes was due to cytotoxicity or decreased proliferation. For that, we used calcein-AM and ethidium homodimer-1 to determine the percentage of viable cells and Ki67 to identify proliferating astrocytes. Hence, we observed a lower number of viable cells at 10 and 50 ng/mL of TNF-α and IFN-γ, respectively, (Figure 4G–H) whereas the proliferative state was unchanged (Figure S3), suggesting that 10 ng/mL reduces cell number by affecting cell viability. Therefore, 10 ng/mL of TNF-α and IFN-γ was chosen for further experiments.

### 3.4. FC Prevents Cytokine-Induced Reactive Astrogliosis

**Figure 5:**
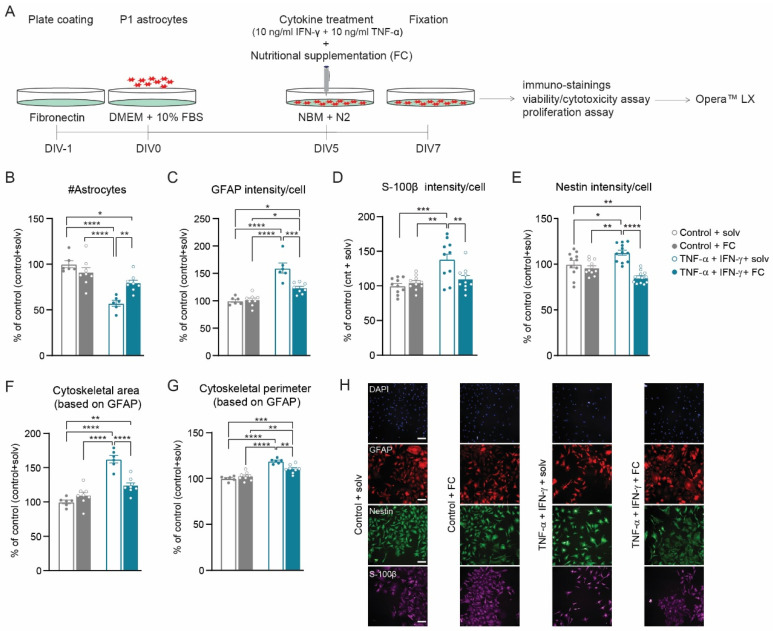
Supplementation with FC (0.05×) prevents the induction of reactive astrogliosis by TNF-α and IFN-γ. (**A**) Experimental design. Analysis of (**B**) number of astrocytes, (**C**) GFAP intensity, (**D**) S-100β intensity, (**E**) Nestin intensity, (**F**) cytoskeletal area (based on GFAP intensity), and (**G**) cytoskeletal perimeter (based on GFAP intensity). (**H**) Representative images of DAPI (blue), GFAP (red), Nestin (green), and S-100β (magenta) for each condition. Data were normalized to control with solvents, which represents a non-stimulated condition (N = 10–18 wells/condition; n = 27,610, n = 33,408, n = 15,792, and n = 29,162 astrocytes analyzed for control + solv, control + FC, TNF-α + IFN-γ + solv, and TNF-α + IFN-γ + FC condition, respectively). Data are presented as the mean ± SEM. One-way ANOVA with a Bonferroni post hoc test: * *p* < 0.05, ** *p* < 0.01, *** *p* < 0.001, and **** *p* < 0.0001. Scale bar: 100 µm.

Next, we aimed to address whether the multi-nutrient combination FC could prevent cytokine-induced reactive astrogliosis, by adding FC at the time of induction (Figure 5A). In addition, FC was tested at two different doses, a low dose (0.05×) and a high dose (0.2×), which is in line with previous work where the addition of FC to a neuron–astrocyte co-culture increased both neuronal survival and postsynaptic terminal maturation in a dose-dependent manner, with an optimal concentration of 0.05 dilution [30]. Supplementation with 0.05× FC prevented reactive astrogliosis, i.e., the cytokine-induced increase in GFAP, S-100β, and Nestin protein levels. In addition, the increase in the astrocyte cytoskeletal area and perimeter were significantly reduced compared to astrocytes treated with solvents (Figure 5B–H). Similar observations were found with high-dose (0.2×) FC (Figure S4A–F), making both doses equally effective in the prevention of reactive astrogliosis. To confirm the observations obtained with AstroScan, we determined total protein levels of GFAP using immunoblotting and, furthermore, included the intermediate filament protein Vimentin, which is also described as a marker of reactive astrogliosis [7]. Indeed, cytokine treatment increased the total protein levels for GFAP and Vimentin, which was prevented by FC (Figure S5). Moreover, FC supplementation prevented the cytokine-induced loss of astrocytes (Figure 5B) by increasing astrocyte viability (Figure S6A,B), whereas it did not affect astrocyte proliferation (Figure S6C,D). Importantly, FC had no effect on astrocyte number (viability and proliferation); intermediate filament cytoskeletal area; and GFAP, S-100β, and Nestin protein levels in control conditions (Figure S6A–D and Fgiure 5B–H). Cytokine and FC treatments also did not affect the expression of Aldh1l1 (Figure S6E,F), an ubiquitous astrocyte protein stably expressed across different models of disease [7,9], showing the specificity of the increased expression of GFAP, S-100β, and Nestin, for the reactive astrogliosis condition. Finally, we determined the nuclear localization of nuclear factor-kappa B (NF-kB), a transcription factor under the control of cytokines [50,51] and a target to revert reactive astrogliosis in several disease models [52,53,54,55]. We found that nuclear localization of NF-kB was not changed in cytokine-treated cultures. However, FC supplementation reduced nuclear NF-kB levels in both control cytokine-treated cultures (Figure S6G,H), in line with a possible role for FC-mediated NF-kB inhibition in the prevention of reactive astrogliosis.

## 4. Discussion

In this study, we used an automated high-throughput method to develop a reactive astrogliosis in vitro model and to determine the effect of nutritional supplementation (FC) on the induction of astrocyte reactivity by pro-inflammatory cytokines. We found that extracellular factors, such as extracellular matrix proteins, have a great influence on the development of the reactive phenotype and that early supplementation with FC prevents the molecular and morphological changes that typically accompany astrocyte reactivity. 

### 4.1. AstroScan: High-Throughput Analysis of Astrocyte Reactivity In Vitro

Molecular and cellular changes of reactive astrocytes have been widely described in different in vitro models [40,56,57,58]. However, the majority of the analyses were performed in a low-throughput manner and for that purpose, we used an automated high content screening system to identify single astrocytes and quantify protein levels and morphological changes. Furthermore, acknowledging the difficulty to characterize such complex phenomena as reactive astrogliosis, we decided to evaluate astrocyte reactivity at different levels: (1) Molecular level; we selected three well-established reactive astrogliosis markers, GFAP, Nestin, and S-100β, which have been described to be upregulated in reactive astrocytes [7,8,9,59,60,61]. More specifically, GFAP and Nestin are intermediate filaments and, therefore, are excellent readouts for cytoskeletal changes, whereas S-100β functions as a signaling molecule and alterations on its expression have been related to a variety of processes related to inflammation [60]. (2) Morphological level; we measured cytoskeletal area and perimeter in order to gain insight in the rearrangement of the cytoskeleton and cell hypertrophy [7,8,9,62]. It should be noted that the observed hypertrophy of the astrocyte intermediate filament cytoskeleton and the upregulation of GFAP, Nestin, and S-100β protein levels are changes also found in reactive astrocytes in vivo. However, for a better understanding of astrocyte reactivity in our assays, future experiments may include functional readouts in parallel to the ones mentioned above [7].

The extracellular microenvironment (ECM) has been reported to play a fundamental role in the cellular response of astrocytes to CNS stimuli [45]. For instance, astrocytes grown on standard culture plates are more reactive than those cultured on ECM coatings [44]. We, therefore, shed light on extracellular factors that contribute to the onset of a cytokine-induced reactive phenotype. Specifically, we investigated which ECM substrates and culture medium were most optimal for the induction of reactive astrogliosis in vitro, i.e., gave the lowest astrocyte reactivity under control conditions with the largest increase under TNF-α and IFN-γ conditions. We found that astrocytes plated on fibronectin showed low reactivity under control conditions and strong reactivity in the presence of TNF-α and IFN-γ. Although astrocytes grown on tenascin-c displayed the most non-activated state in the control conditions, which is in line with previous observations [44], cytokine treatment failed to induce astrocyte reactivity. Finally, PDL/laminin caused high levels of astrocyte reactivity under basal conditions and cytokine treatment was not able to induce this further. These observations are in line with published studies reporting that the astrocyte-cytokine response is influenced by the astrocyte interaction with ECM molecules [45,63,64]. It should be noted that fibronectin is present in low amounts in the parenchyma from healthy adult human tissue. However, its expression is upregulated in CNS injuries that involve scar formation and inflammation [65,66,67,68,69]. Thus, the reported effects of fibronectin on astrocyte reactivity serve as a tool to develop a reactive astrogliosis in vitro model and may provide insight into the role of astrocyte–ECM interaction in the context of cytokine-induced reactive astrogliosis.

Similarly, the impact of culture media on astrocyte reactivity was evaluated since its composition has a direct effect on the astrocytic response to specific stimuli [47,70,71]. Astrocytes showed the most robust reactive phenotype upon TNF-α and IFN-γ stimulation when added in serum-free media (NBM supplemented with B27 or N2) compared to serum-containing media. Finally, we showed that 10 ng/mL of TNF-α and IFN-γ is sufficient to reduce astrocyte viability and induce astrocyte reactivity, which is in line with previous reports [12,42,43,72,73].

Taken together, we set up AstroScan to measure astrocytic molecular and morphological changes in a high-throughput manner and used this to develop a cytokine-induced reactive astrogliosis model optimized for ECM composition and culture media. This work could help to identify astrocyte-specific therapeutic targets as well as to enable high-throughput testing of drugs directed to reactive astrocytes. 

### 4.2. FC Supplementation Prevents Cytokine-Induced Reactive Astrogliosis

Nutritional supplementation with FC has previously been shown to exert neuroprotective and neurorestorative effects in various models of neurodegenerative disorders and acute CNS injury characterized by neuroinflammation and reactive glial responses [37,74,75,76,77]. Using AstroScan and our current in vitro model for reactive astrocytes, we found that treatment with FC has an acute beneficial effect on cytokine-induced reactive astrogliosis. Indeed, supplementation with FC prevented the cytokine-induced upregulation of GFAP, Vimentin, S-100β, and Nestin protein levels and cytoskeletal hypertrophy. In addition, FC prevented the cytokine-induced reduction of astrocyte numbers, probably via its interference with cytokine-induced cell death. Interestingly, FC had no effect on these parameters under non-stimulated conditions, which is in line with our previous work on hippocampal neuron–astrocyte co-cultures where FC did increase neuronal survival and postsynaptic maturation but had no effect on the number of astrocytes or on GFAP protein levels and astrocyte morphology, all in the absence of pro-inflammatory cytokines [30]. A direct effect of FC on cytokine-induced reactive astrogliosis has so far not been reported. Nevertheless, our findings are in line with Pallier et al. (2015), where rats kept on an FC-containing diet for nine weeks after compression of the spinal cord showed reduced macrophage recruitment at the site of injury and decreased reactive astrogliosis (assessed by GFAP protein levels). Our observation that FC reduces nuclear localization of the transcription factor NF-kB provides a first step toward understanding the molecular mechanism by which dietary intervention with FC attenuates reactive astrogliosis. Activation of NF-kB plays an important role in the transcriptional activation of pro-inflammatory cytokines and intermediate filaments [78], whereas inhibition of NF-kB reduces reactive astrogliosis in several disease models [52,53,54,55]. Although NF-kB has been demonstrated to be activated by cytokines, including TNF-α and IFN-γ [50,51], we did not detect increased NF-κB nuclear localization in reactive astrocytes, which may be due to the fact that cytokine-induced NF-kB activation is transient and can no longer be detected 2 days after treatment [79,80]. Overall, our data indicate that FC may prevent reactive astrogliosis by reducing the activity of NF-kB.

Interestingly, anti-inflammatory effects of individual components of FC have also been described. DHA and EPA are known to bind to PPARγ, which in turn inhibits NF-kB nuclear translocation and, thus, attenuates inflammatory response [81,82]. In fact, acute intravenous injection of DHA and EPA may reduce astrocyte reactivity and neuroinflammation and promote recovery of the injured CNS [50,83]. Furthermore, vitamin B12 and B9 deficiency causes increased oxidative stress, upregulated GFAP and Vimentin protein levels, and hypertrophy of the astrocyte intermediate filament cytoskeleton in vitro [68,69]. Vitamins B12 and B9 are both present in FC and may contribute to the observed prevention of gliosis by FC. Although these studies uncover an important role of omega-3 polyunsaturated fatty acids (PUFAs) and specific vitamins in reducing reactive astrogliosis and improving brain health, a large number of preclinical studies have reported that specific nutrient combinations rather than a single component can act synergistically and improve therapeutic outcomes [37,84,85].

Hence, a multi-nutrient dietary approach that can revert reactive astrogliosis is of high significance from a translational perspective. Long-term dietary intervention with FC in the early stages of AD is both feasible and effective in supporting brain network connectivity and memory performance [33,34,35]. Here, we demonstrate, for the first time, that FC is able to directly prevent reactive astrogliosis when induced with pro-inflammatory cytokines TNF-α and IFN-γ. Thus, in the context of neuroinflammation such as AD, inhibition of reactive astrogliosis with FC may contribute to the observed positive effects of FC in terms of slowing functional decline, brain atrophy, and disease progression [31,32] and possibly be relevant for other CNS diseases.

Further research is needed in order to better understand the molecular mechanisms underlying the beneficial effects of FC on reactive astrogliosis in vitro and in vivo and how these insights might be turned into an advantage for the treatment of neurodegenerative diseases.

## Figures and Tables

**Table 1:** FC composition. Final concentrations of FC components (µM) for the two conditions: 0.2× FC (high dose) and 0.05× FC (low dose).

	0.2× FC (µM)	0.05× FC (µM)
DHA	2.88	0.72
EPA	2.02	0.51
Uridine	10	2.5
Choline	4	1
Vitamin B6	2	0.5
Vitamin B12	0.02	0.005
Vitamin B9	3	0.75
PC	5	1.25
Vitamin C	15	3.75
Vitamin E	4	1
Selenium	0.02	0.005

Docosahexaenoic acid, DHA; eicosapentaenoic acid, EPA; and phosphatidylcholine, PC.

## Supplementary Materials

The following supporting information can be downloaded at https://www.mdpi.com/article/10.3390/cells11091428/s1, Figure S1: High-purity astrocyte cultures seven days after isolation; Figure S2: Cytokine-induced reactive astrogliosis model: 24 h induction; Figure S3: Cytokine-induced reactive astrogliosis does not affect astrocyte proliferation; Figure S4: Supplementation with high dose FC (0.2×) prevents the induction of reactive astrogliosis by TNF-α and IFN-γ; Figure S5: FC prevents the cytokine-induced increase in GFAP and Vimentin protein levels; Figure S6: The effect of cytokine treatment and FC supplementation on astrocyte viability, proliferation, Aldh1l1 protein levels and NF-κB nuclear translocation.
