# Supplementary material for: High-Throughput Analysis of Astrocyte Cultures Shows Prevention of Reactive Astrogliosis by the Multi-Nutrient Combination Fortasyn Connect
Source: Cells. 2022 Apr 22;11(9):1428. doi: 10.3390/cells11091428 (PMC9099974; doi:10.3390/cells11091428)
Supplement: Supplementary file 1 [file cells-11-01428-s001.zip › cells-1396586-supplementary.pdf]

Supplementary Materials; Badia-Soteras et al

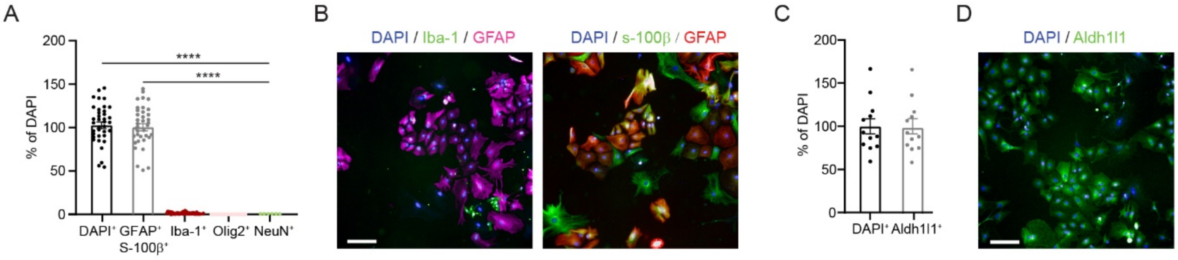

Figure S1. High-purity astrocyte cultures seven days after isolation. A) Quantification of astrocytes (GFAP+/S-100β+), microglia (Iba-1+), oligodendrocytes/OPCs (Olig2+) and neurons (NeuN+) relative to all DAPI+ cells/well. N=36-38 wells, n=50433 (DAPI), n=45528 (astrocytes), n=780 (microglia), n=17 (oligodendrocytes). Data is presented as mean ± SEM. One-way ANOVA with Bonferroni post hoc test: ****$p<0.0001$. B) Representative images of the primary astrocyte culture. Note that 98% of the Dapi+ cells are astrocytes and most of the remaining 2% are microglia. C) Quantification of DAPI+ cells expressing Aldh1l1, an astrocyte-specific marker. N=12 wells/condition, n=26130 (DAPI), n=25871 (Aldh1l1). D) Representative images of Aldhl1l-expresing astrocytes. Data is presented as mean ± SEM. Unpaired t-test. Scale bar: 100 μm.

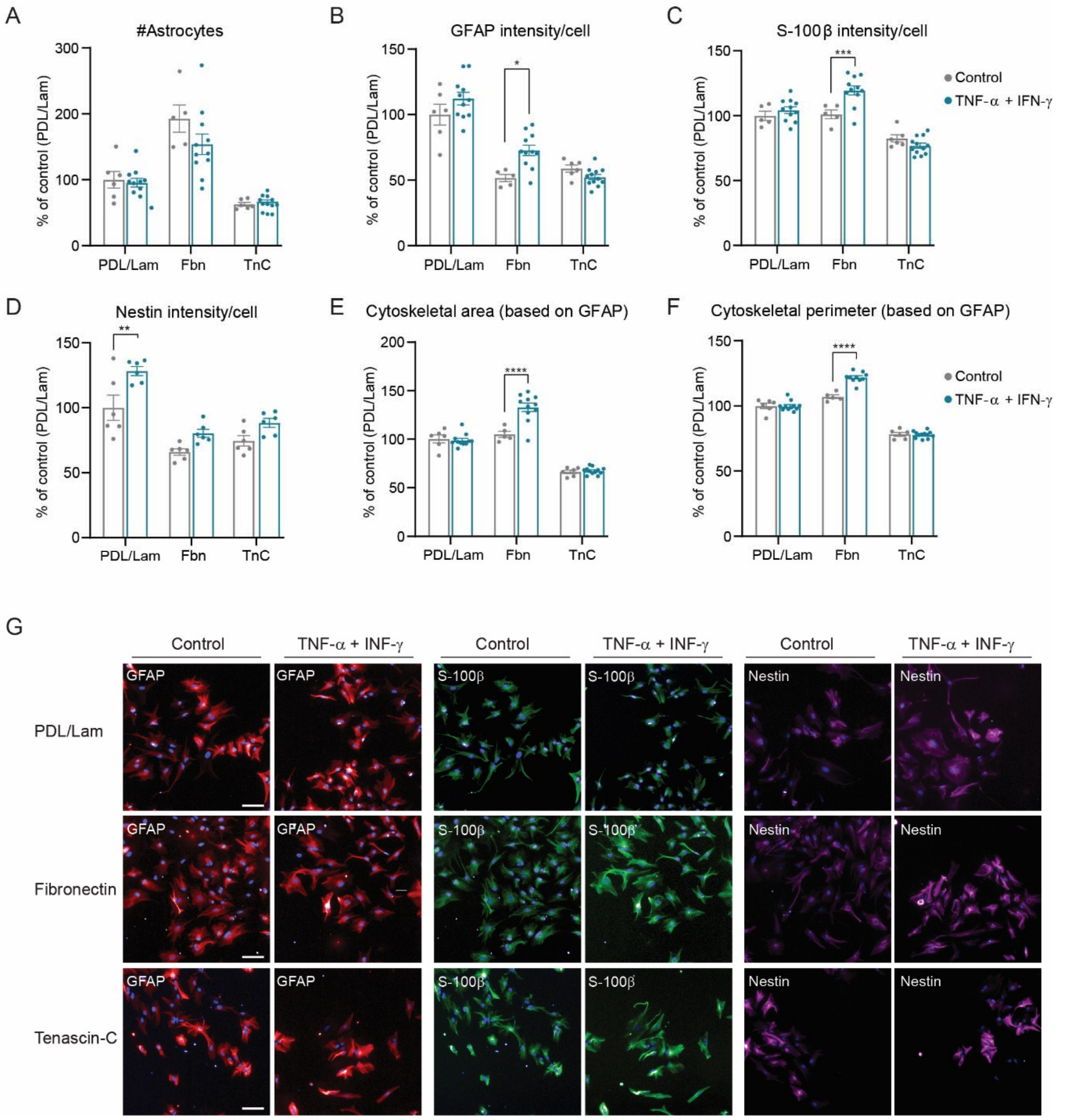

Figure S2. Cytokine-induced reactive astrogliosis model: 24 hours induction. Analysis of (A) number of astrocytes, (B) GFAP intensity per cell, (C) S-100β intensity per cell, (D) Nestin intensity per cell, (E) cytoskeletal area (based on GFAP intensity) and (F) cytoskeletal perimeter (based on GFAP intensity). G) Representative images of DAPI (blue), GFAP (red), S-100β (green) and Nestin (magenta) for each condition. Data was normalized to PDL/Lam unstimulated. N=5-10 wells/condition), n=4438 (control PDL/Lam), n=7126 (control Fbn), n=2780 (control TnC), n=7804 (TNF-α + IFN-γ PDL/Lam), n=12533 (TNF-α + IFN-γ Fbn), n=5486 (cytokines TnC). Data is presented as mean ± SEM. Two-way ANOVA with Bonferroni post hoc test: *p<0.05, **p<0.01, ***p<0.001, ****p<0.0001. Scale bar: 100 μm.

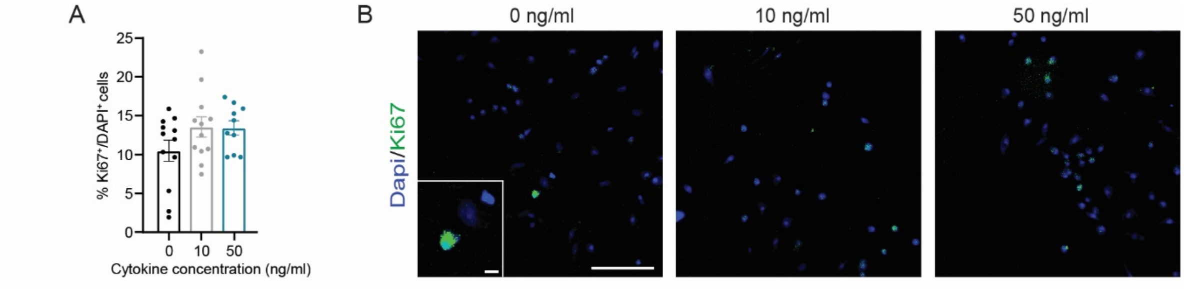

Figure S3. Cytokine-induced reactive astrogliosis does not affect astrocyte proliferation. A) Analysis of $Ki67^+$ astrocyte nuclei upon incremental cytokine concentration, the number of $Ki67^+$ nuceli is given as percentage of the total number of DAPI+ nuclei. B) Representative images of DAPI (blue) and Ki67 (green). Scale bar: 100 µm (overview) and 10 µm (inset). Data was normalized to control condition (0 ng/ml). N=10-12 wells/condition, n=5077 (0 ng/ml), n=3576 (10 ng/ml), n=2606 (50 ng/ml). Data is presented as mean ± SEM. One-way ANOVA with Bonferroni post hoc test.

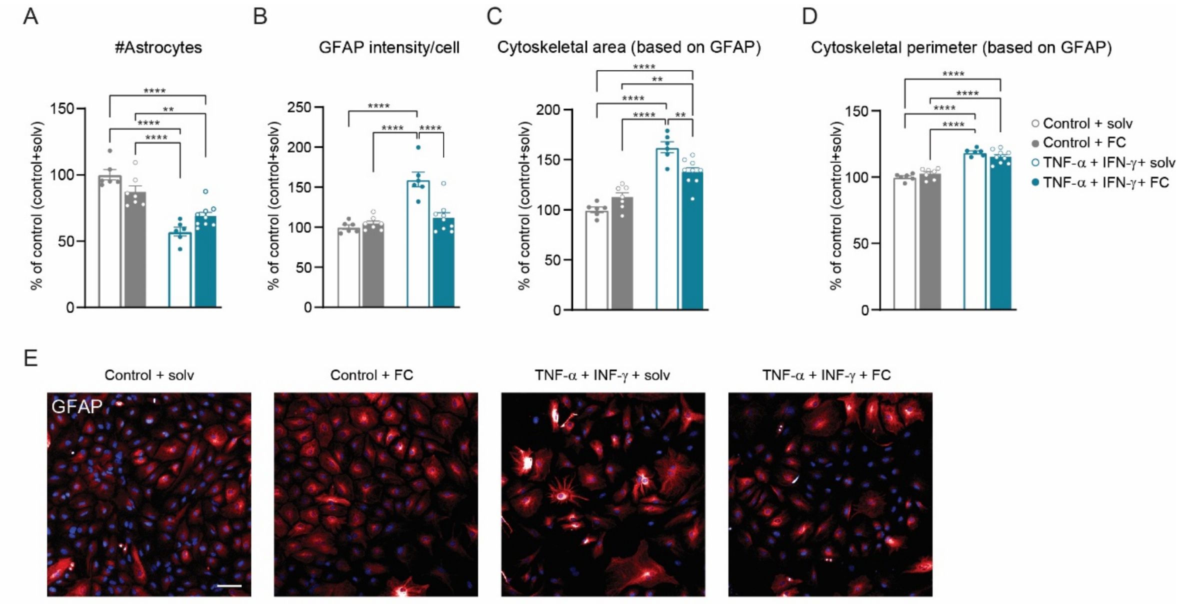

Figure S4. Supplementation with high dose FC (0.2x) prevents the induction of reactive astrogliosis by TNF-α and IFN-γ. Analysis of (A) number of astrocytes, (B) GFAP intensity, (C) cytoskeletal area (based on GFAP intensity) and (D) cytoskeletal perimeter (based on GFAP intensity). E) Representative images of DAPI (blue) and GFAP (red) for each condition. Data was normalized to control with solvents, which represents a non-stimulated condition. N=10-18 wells/condition, n=27610 (control + solv), n=28034 (control + FC), n=15792 (TNF-α + IFN-γ + solv), n=28518 (TNF-α + IFN-γ + FC). Data is presented as mean ± SEM. Two-way ANOVA with Bonferroni post hoc test: *$p<0.05$, **$p<0.01$, ***$p<0.001$, ****$p<0.0001$. Scale bar: 100 μm.

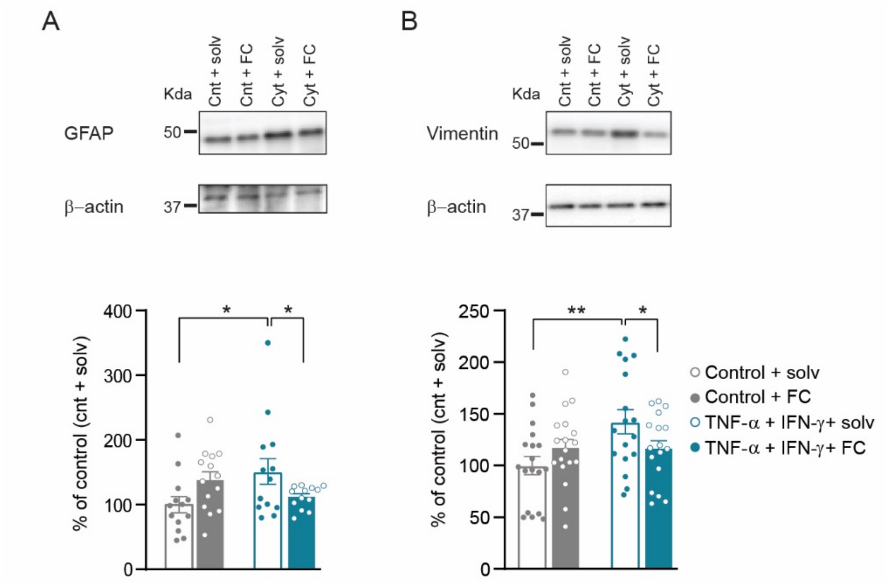

Figure S5. FC prevents the cytokine-induced increase of GFAP and Vimentin protein levels. Immunoblotting for (A) GFAP and (B) Vimentin. Total protein levels for GFAP and Vimentin were normalized to β-actin. Data was normalized to control with solvents (N=13-18 wells/condition from 3 independent experiments). Data is presented as mean ± SEM. One-way ANOVA with Bonferroni post hoc test: *$p<0.05$, **$p<0.01$.

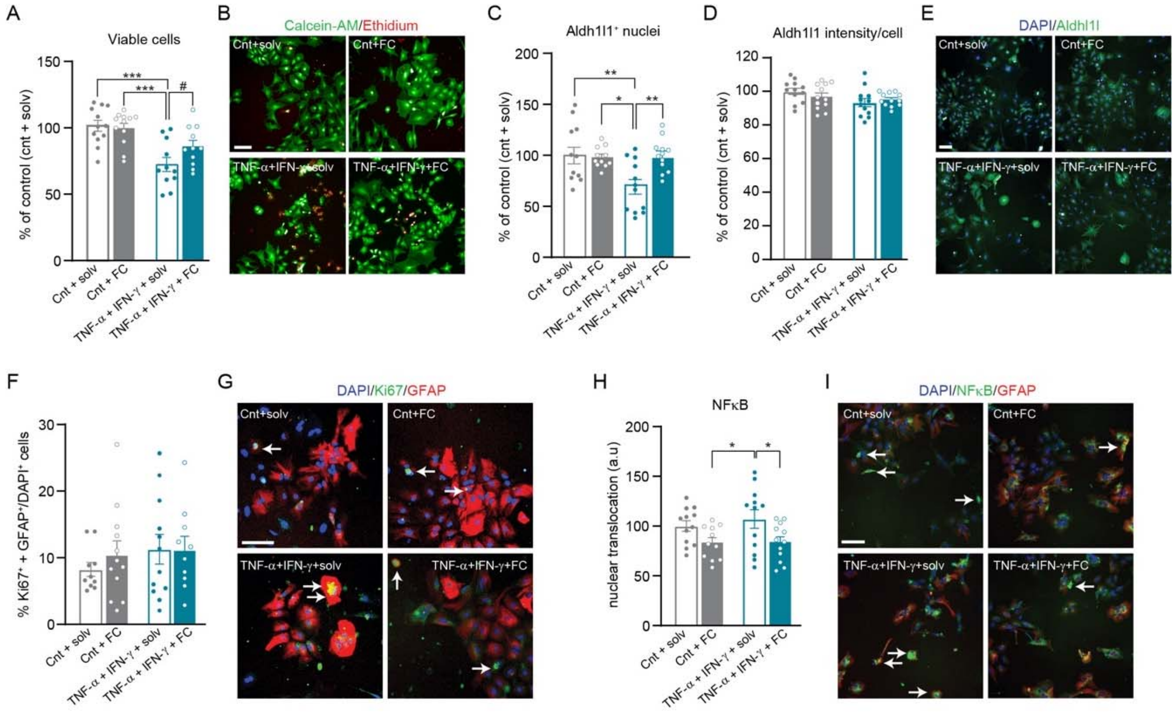

Figure S6. The effect of cytokine treatment and FC supplementation on astrocyte viability, proliferation, Aldh1l1 protein levels and NF-κB nuclear translocation. A) Quantification of viable cells upon cytokine stimulation and FC supplementation. N=11-12 wells/condition, n=37341 (control + solv), n=35815 (control + FC), n=23609 (TNF-α + IFN-γ + solv), n=31306 TNF-α + IFN-γ + FC. B) Representative images of Calcein-AM (alive cells) and Ethidium homodimer-1 (dead cells). C) Percentage of Aldh1l1$^+$ astrocytes upon cytokine stimulation and FC supplementation. N=10-12 wells/condition, n= 6241 (control + solv), n=5899 (control + FC), n=4608 (TNF-α + IFN-γ + solv), n=6026 (TNF-α + IFN-γ + FC). D) Quantification of Aldh1l1 signal intensity upon cytokine stimulation and FC. N=10-12 wells/condition, n= 6241 (control + solv), n=5899 (control + FC), n=4608 (TNF-α + IFN-γ + solv), n=6026 (TNF-α + IFN-γ + FC). E) Representative images of DAPI and Aldh1l1. F) Quantification of Ki67$^+$ proliferating astrocytes (expressing GFAP) upon cytokine stimulation and FC. N=10-12 wells/condition, n=10311 (control + solv), n=9525 (control + FC), n=9025 (TNF-α + IFN-γ + solv), n=9722 (TNF-α + IFN-γ + FC). G) Representative images of proliferating and non-proliferating astrocytes. White arrows indicate Ki67$^+$ proliferating astrocytes. H) Quantification of the NF-κB nuclear translocation. N=10-12 wells/condition, n=43558 (control + solv), n=38540 (control + FC), n=34726 (TNF-α + IFN-γ + solv), n=26766 (TNF-α + IFN-γ + FC). I) Representative images of GFAP-expressing astrocytes and NFκB. White arrows represent nuclear translocation of NFκB. Data is presented as mean ± SEM. One-way ANOVA with Bonferroni post hoc test: *$p<0.05$, **$p<0.01$, ***$p<0.001$, #$p<0.1$.
